# Cell-type specific short-term plasticity at auditory nerve synapses controls feed-forward inhibition in the dorsal cochlear nucleus

**DOI:** 10.3389/fncir.2014.00078

**Published:** 2014-07-04

**Authors:** Miloslav Sedlacek, Stephan D. Brenowitz

**Affiliations:** Section on Synaptic Transmission, National Institute on Deafness and Other Communication Disorders, National Institutes of HealthBethesda, MD, USA

**Keywords:** dorsal cochlear nucleus, auditory nerve, synaptic transmission, synaptic plasticity, feedforward inhibition

## Abstract

Feed-forward inhibition (FFI) represents a powerful mechanism by which control of the timing and fidelity of action potentials in local synaptic circuits of various brain regions is achieved. In the cochlear nucleus, the auditory nerve provides excitation to both principal neurons and inhibitory interneurons. Here, we investigated the synaptic circuit associated with fusiform cells (FCs), principal neurons of the dorsal cochlear nucleus (DCN) that receive excitation from auditory nerve fibers and inhibition from tuberculoventral cells (TVCs) on their basal dendrites in the deep layer of DCN. Despite the importance of these inputs in regulating fusiform cell firing behavior, the mechanisms determining the balance of excitation and FFI in this circuit are not well understood. Therefore, we examined the timing and plasticity of auditory nerve driven FFI onto FCs. We find that in some FCs, excitatory and inhibitory components of FFI had the same stimulation thresholds indicating they could be triggered by activation of the same fibers. In other FCs, excitation and inhibition exhibit different stimulus thresholds, suggesting FCs and TVCs might be activated by different sets of fibers. In addition, we find that during repetitive activation, synapses formed by the auditory nerve onto TVCs and FCs exhibit distinct modes of short-term plasticity. Feed-forward inhibitory post-synaptic currents (IPSCs) in FCs exhibit short-term depression because of prominent synaptic depression at the auditory nerve-TVC synapse. Depression of this feedforward inhibitory input causes a shift in the balance of fusiform cell synaptic input towards greater excitation and suggests that fusiform cell spike output will be enhanced by physiological patterns of auditory nerve activity.

## Introduction

In many regions of the mammalian brain, feed-forward inhibition (FFI) represents a complex synaptic arrangement in neuronal networks that results from parallel activation of principal cells and inhibitory interneurons by the same excitatory input (Buzsaki, 1984; Pouille and Scanziani, 2001; Blitz and Regehr, 2005; Gabernet et al., 2005; Mittmann et al., 2005; Cruikshank et al., 2007; Torborg et al., 2010; Ellender et al., 2011; Kuo and Trussell, 2011; Najac et al., 2011; Zhou et al., 2012). Activation of inhibitory interneurons consequently provides inhibition to principal cells to reduce their excitability. The temporal resolution of integration of synaptic inputs depends on the time window within which excitatory inputs can be summated and reach the threshold for firing an action potential in the postsynaptic neuron (Pouille and Scanziani, 2001). The precise timing of excitation and inhibition plays a significant role during high frequency repetitive neuronal activity and has been shown previously to control short-term synaptic plasticity of excitatory and inhibitory inputs (Gabernet et al., 2005; Torborg et al., 2010).

The dorsal cochlear nucleus (DCN) integrates non-auditory and auditory information and plays a role in localization of sound sources and filtering self-generated noise (Shore and Zhou, 2006; Requarth and Sawtell, 2011). Fusiform cells (FCs) are the principal neurons of the DCN that integrate multiple excitatory and inhibitory synaptic inputs onto their apical and basal dendrites (Voigt and Young, 1980, 1990; Blackstad et al., 1984; Oertel and Wu, 1989; Berrebi and Mugnaini, 1991; Zhang and Oertel, 1994). Excitatory inputs contacting apical dendrites of FCs come from granule cell parallel fibers located in the superficial molecular layer. These fibers also innervate cartwheel cells, local glycinergic interneurons that also provide inhibition to FC apical dendrites (Roberts and Trussell, 2010; Kuo and Trussell, 2011). Excitatory inputs onto basal dendrites are conveyed via auditory nerve fibers that carry precisely timed, tonotopically organized acoustic information. Additional excitatory input is formed by the T-stellate cells that send their axons from the ventral cochlear nucleus to the deep layer of the DCN (Oertel and Young, 2004; Oertel et al., 2010). Predominantly glycinergic inhibition terminating onto the basal dendrite of FCs is represented by inputs from the tuberculoventral cells (TVCs), also referred to as vertical cells, and D-stellate cells (Zhang and Oertel, 1994), which share the same auditory nerve input with FCs. This complex synaptic arrangement associated with the basal dendrite forms the basis for a feed-forward inhibitory circuit associated with transmission of acoustic information via FCs. Moreover, TVCs form inhibitory synapses onto each other (Kuo et al., 2012). TVCs lie in bands parallel to isofrequency laminae, and their targets, including FCs in the DCN, are innervated by the same auditory nerve fibers (Wickesberg and Oertel, 1988; Voigt and Young, 1990). Moreover, TVCs are sensitive to narrowband stimuli, as only a small number of auditory nerve fibers provide excitation to these interneurons, distinguishing them from D-stellate cells that are sensitive to broadband sounds and are innervated by auditory nerve fibers tuned to a wider frequency range (Voigt and Young, 1990; Winter and Palmer, 1995; Palmer and Winter, 1996). Therefore, inhibition of FCs by TVCs can regulate firing behavior of FCs (Nelken and Young, 1994; Oertel and Young, 2004), although recent evidence indicates the strength of individual connections might be rather weak (Kuo et al., 2012).

To examine the basis for feed-forward inhibitory control and plasticity of auditory processing in the DCN, we determined the synaptic mechanisms that control the balance of excitation and inhibition and affect the output from the nucleus. In this study, we show that short-term synaptic plasticity of auditory nerve-evoked disynaptic inhibition onto FCs exhibits facilitation when activated directly by stimulating inhibitory inputs onto FCs, similar to what has been shown previously using paired recordings from fusiform and TVCs (Kuo et al., 2012). In addition, we show that short-term synaptic plasticity, that is cell type specific in this synaptic circuit, controls FFI received by FCs. We demonstrate that facilitation of TVC-mediated inhibition of FCs shifts to significant depression when driven by the auditory nerve. This shift in synaptic plasticity and excitation-inhibition balance in FCs during repetitive auditory nerve stimulation results from pronounced activity-dependent short-term depression of auditory nerve synapses onto TVCs.

## Materials and methods

### Cochlear nucleus slice preparation

All experiments were conducted in accordance with animal protocols approved by the NIH Animal Care and Use Committee. P17–P22 C57BL/6 mice of either sex were deeply anesthetized with isoflurane before decapitation and parasagittal brainstem slices containing the cochlear nucleus were cut using a ceramic blade mounted on a vibrating microtome (Leica VT1200S, Leica Microsystems). In order to preserve the complex circuitry and long-distance synaptic connections necessary to study FFI, a midline cut was made, the brainstem was cut into two halves and the medial surface of the right half was glued down to the slicing platform. Then, the first cut was made right above the lateral surface of the cochlear nucleus without touching the surface of the nucleus or the auditory nerve root. A second cut was made to obtain a thick (380–450 μm) slice containing most of the cochlear nucleus. Using a thick slice preparation allowed us to preserve the auditory nerve inputs to the DCN which is critical for studying disynaptic inhibition. Dissections were performed in an ice-cold, sucrose-based extracellular solution that contained the following (in mM): 75 NaCl, 26 NaHCO_3_, 75 sucrose, 25 glucose, 2.5 KCl, 1.25 NaH_2_PO_4_, 7 MgCl_2_, 0.5 CaCl_2_, 2 Na-pyruvate, 3 myo-inositol, 0.4 Na-ascorbate (pH 7.35, ~325 mOsm). Slices were then incubated in the same solution for 20 min at 34°C, transferred to saline solution that contained the following (in mM): 125 NaCl, 26 NaHCO_3_, 25 glucose, 2.5 KCl, 1.25 NaH_2_PO_4_, 1 MgCl_2_, 2 CaCl_2_, 2 Na-pyruvate, 0.4 Na-ascorbate (pH 7.35, ~315 mOsm) and were incubated for additional 20 min at 34^°^C. All solutions were bubbled with 5% O_2_/95% CO_2_.

### Electrophysiology

Slices were placed in a recording chamber in a way that the intact lateral surface of the nucleus faced the bottom of the chamber and all recordings were made from the medial surface of the DCN. Slices were continuously perfused (2–3 ml/min) with saline extracellular solution. Fusiform and TVCs were visually identified using a 60 × 0.9 NA objective (Olympus) and infrared differential interference contrast. Recording electrodes (2.2–4 MΩ) pulled from thick-walled borosilicate glass (Sutter Instruments) were filled with intracellular solution that contained (in mM): 120 CsMeSO_4_, 10 HEPES, 5 NaCl, 3 MgSO_4_, 2 QX-314, 4 Mg-ATP, 0.4 Na-GTP, 14 Tris-phosphocreatine for voltage-clamp experiments, or (in mM): 125 KMeSO_4_, 10 HEPES, 5 NaCl, 1 MgCl_2_, 4 Mg-ATP, 0.4 Na-GTP, 14 Tris-phosphocreatine for current-clamp experiments. To verify the identity of recorded fusiform and tuberculoventral neurons, all intracellular solutions were supplemented with Alexa Fluor 594 hydrazide (20 μM). Cell morphology was visualized using a two-photon laser scanning microscope and a Ti:sapphire pulsed laser (Chameleon, Coherent) tuned to 840 nm for excitation. Data were filtered at 3 or 6 kHz using a Multiclamp 700B amplifier (Molecular Devices) and sampled at 10 or 20 kHz, respectively. Series resistance (7–18 MΩ) was compensated by 75% and experiments in which the series resistance increased by >20% were excluded from further analysis. To evoke synaptic responses, a tungsten bipolar stimulating electrode with 140 μm tip spacing and with tips bent at a 45° angle (FHC, Bowdoin, ME) was placed in the auditory nerve root (for FFI and direct stimulation of excitatory inputs), or in the deep layer of the DCN (for direct stimulation of inhibitory inputs). Because FCs also receive excitatory granule cell inputs in addition to the auditory nerve inputs, care was taken to directly stimulate auditory nerve fibers in region of the nerve root attached to the ventral region of the cochlear nucleus. The nerve root was readily identified with transmitted light under a 4x objective, as well as auditory nerve fibers within the root, and could be visually traced beyond the ascending/descending auditory nerve branch bifurcation. However, the placement of the stimulating electrode within the auditory nerve root did not prevent the activation of T-stellate cell excitatory inputs onto FCs in some cases, which could be seen as disynaptic excitation in fusiform cell recordings (for example see Figure 2A). We observed the disynaptic excitation in ~35% of FCs while recording auditory nerve (AN) evoked EPSCs. These recordings were used for further analyses since the later, presumably T-stellate cell-mediated peak did not interfere with the first peak in any way. Moreover, during the FFI trials, the fast kinetics and fast onset of AN evoked inhibitory post-synaptic currents (IPSCs) eliminated the T-stellate cell mediated peak completely. To further ensure and verify that excitation of FCs originated from activation of the auditory nerve fibers, we tested short-term synaptic plasticity of the recorded excitatory responses and verified that excitatory responses were evoked by stimulation of the auditory nerve inputs by the presence of short-term synaptic depression. In contrast. stimulation of parallel fiber synapses evoked synaptic responses that strongly facilitate (Tzounopoulos et al., 2004; Roberts and Trussell, 2010) and can therefore be distinguished from auditory nerve stimulation. Synaptic responses were evoked with 0.2 ms current pulses (0–100 μA) delivered by an isolated stimulus unit (World Precision Instruments). To record FFI consisting of an EPSC-IPSC sequence, FCs were voltage clamped at −40 mV, a holding potential between the reversal potentials for excitatory and inhibitory transmission, and no synaptic blockers were added to the perfusion solution. Direct auditory nerve-evoked EPSCs were recorded at −60 mV with strychnine (2 μM) and picrotoxin (40 μM) in the bath. Direct IPSCs were recorded at −40 mV with 6,7-dinitrodihydroquinoxaline-2,3-dione (DNQX) (20 μM) and R-CPP (5 μM) in the bath. To analyze timing of FFI, EPSCs were recorded at the chloride reversal potential (E_Cl_ = −59 to −65 mV), IPSCs were recorded at the reversal potential for glutamatergic transmission (E_glu_ = +5 to +12 mV), and no synaptic blockers were present in the bath. Recordings were not corrected for the liquid junction potential. Picrotoxin was from Tocris Cookson, DNQX and R-CPP were from Abcam, Alexa Fluor 594 hydrazide was from Invitrogen, all other chemicals were from Sigma. All recordings were performed at 33–35^°^C.

**Figure 1:**
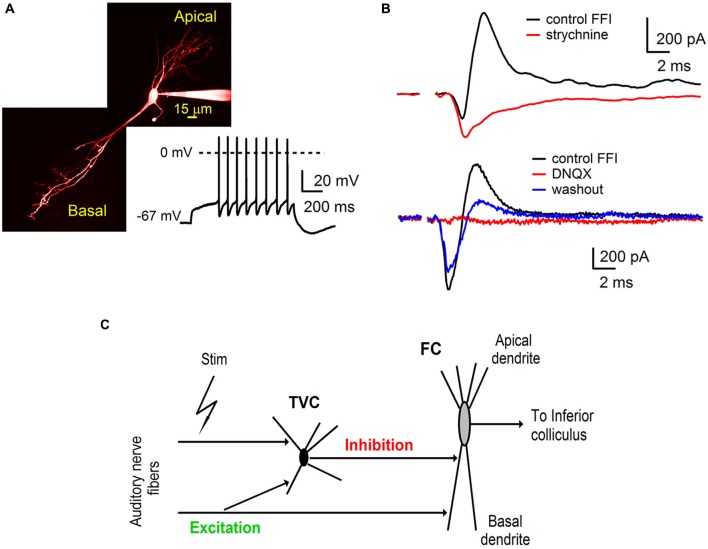
**Auditory nerve drives feed-forward inhibition onto fusiform cells in the DCN. (A)** A two-photon laser scanning microscopy image of a fusiform cell loaded with Alexa Fluor 594 showing apical and basal dendritic branching. The trace shows a representative example of firing properties of fusiform cells (FCs). Note the delayed spiking pattern (build up) while the cell was held at hyperpolarized membrane potentials. **(B)** Voltage clamp recordings of feed-forward inhibition from FCs evoked by extracellular stimulation of the auditory nerve. Inhibitory component of the EPSC-IPSC sequence (outward deflection of the black trace) was completely blocked with 2 μM strychnine (top traces, *n* = 3 cells). Application of AMPA receptor antagonist 6,7-dinitrodihydroquinoxaline- 2,3-dione (DNQX) (20 μM) reversibly blocked both components of the sequence (bottom traces, *n* = 5 cells). Stimulation artifacts were removed for clarity. Traces represent averages of 5–10 trials. **(C)** A schematic of the feed-forward inhibitory circuit driven by the auditory nerve in the dorsal cochlear nucleus. Auditory nerve fibers synapse onto FC, as well as onto tuberculoventral cells (TVC) which further provide inhibition to the FC.

**Figure 2:**
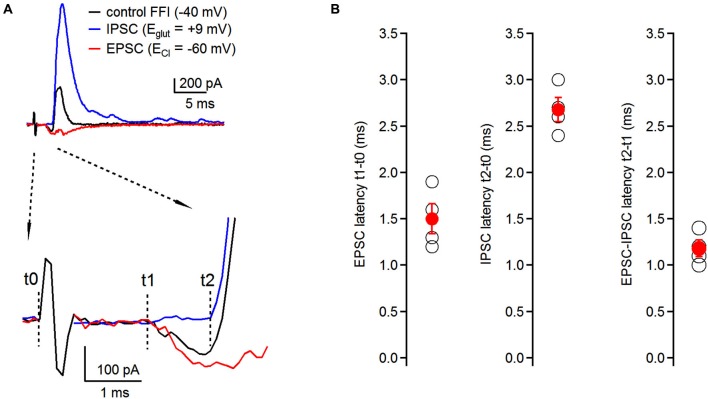
**Timing of feed-forward inhibition onto fusiform cells. (A)** Latencies of individual components of the EPSC-IPSC sequence (black trace) were examined at respective reversal potentials for excitatory and inhibitory transmission. EPSCs (red trace) were recorded at E_Cl_ (−60 ± 0.7 mV, *n* = 4 cells); IPSCs (blue trace) were recorded at E_glut_ (8.25 ± 1.5 mV, *n* = 4 cells) (top traces). No synaptic blockers were added to the perfusion solution. Timing of individual components was analyzed using the time difference between the beginning of the stimulating artifact (t0) and the onset of synaptic events (t1 for EPSCs, t2 for IPSCs). EPSC-IPSC latency was calculated as t2 − t1 (bottom traces). Traces represent averages of 5–10 trials. Only stimulation artifact in the control FFI (black) trace is shown, the other two were removed from EPSC and IPSC traces for clarity. **(B)** Plots of latencies of EPSCs and IPSCs from the stimulation artifact, and timing of EPSC-IPSC sequence. Each black data point represents one neuron; red data points represent averages ± SEM; *n* = 4 cells.

### Data analysis

All data were acquired and analyzed using custom routines written in Matlab (MathWorks) and IgorPro (WaveMetrics), respectively. Averages are presented as means ± SEM. To distinguish between facilitating and depressing synapses, the ratio of the 10th stimulus to the 1st stimulus (S10/S1) was calculated. Recordings with S10/S1>1 were considered facilitating, while those with S10/S1<1 were considered to be depressing. Latencies were calculated as the time between the beginning of the stimulus artifact and the onset of excitatory/inhibitory synaptic event.

## Results

### Auditory nerve activity triggers feed-forward inhibition in fusiform cells

Basal dendrites of FCs in the DCN receive direct excitatory inputs from the auditory nerve and inhibitory synaptic inputs from TVCs in the deep layer of the DCN (Zhang and Oertel, 1993; Rhode, 1999; Oertel and Young, 2004; Kuo et al., 2012). To further investigate the roles of synaptic excitation and inhibition in auditory processing by FCs, we used parasagittal slices of the cochlear nucleus (see Materials and Methods) that preserve components of the synaptic circuit associated with the fusiform cell basal dendrites (Figure 1).

Whole-cell voltage clamp recordings were made from visually identified FCs. The membrane potential of the fusiform cell was clamped at −40 mV, which is above the reversal potential for inhibitory synaptic currents (E_Cl_, ~−60 mV) and below the reversal for excitatory synaptic currents (E_Glu_, ~0 mV). Synaptic responses were evoked from a distance of several hundred micrometers from the target postsynaptic neurons. Stimulation of auditory nerve fibers evoked postsynaptic currents in FC (Figure 1A) that consisted of a sequence of inward excitatory (EPSC) and outward inhibitory (IPSC) components (Figure 1B). Subsequent bath application of the glycine receptor antagonist strychnine (2 μM) completely blocked the outward component of the synaptic current (Figure 1B, top *n* = 3 cells). These results indicate that the inward EPSCs recorded from the FC were evoked directly by stimulation of the auditory nerve fibers.

In separate experiments, application of the AMPA receptor antagonist DNQX (20 μM) completely abolished both the inward and outward component. The blockade was partially reversible (Figure 1B, bottom *n* = 5 cells). Sensitivity of inhibitory synaptic transmission to a blocker of excitatory transmission demonstrates that the IPSCs were evoked by auditory nerve stimulation rather than by direct activation of inhibitory fibers and were therefore disynaptic in nature. Interneurons providing inhibition to FCs in the deep layer of the DCN have been identified as TVCs (Zhang and Oertel, 1993; Rhode, 1999; Oertel and Young, 2004; Kuo et al., 2012). We conclude that auditory nerve activity drives a feed-forward inhibitory circuit in the DCN that includes tuberculoventral cells, DCN interneurons that provide inhibition to FCs in the deep layer of the DCN.

### Timing of feed-forward inhibition onto basal dendrites of fusiform cells

Timing of excitatory and inhibitory inputs can have significant consequences for the generation of action potentials in postsynaptic neurons both in the auditory system (Oertel, 1999; Brand et al., 2002), as well as in other brain regions (Buzsaki, 1984; Gil and Amitai, 1996; Borg-Graham et al., 1998). Therefore, having demonstrated the presence of disynaptic inhibition onto fusiform cells evoked by auditory nerve stimulation, we next examined the relative timing of individual components of the feed-forward EPSC-IPSC sequence. We recorded synaptic responses from fusiform cells, and by voltage clamping the cells at different holding potentials we isolated individual components without having to use pharmacological tools. First, we recorded the control FFI sequence at −40 mV with both inward and outward components present (Figure 2A, top). To isolate the inhibitory component of the sequence, fusiform cells were voltage clamped at the reversal potential for excitatory transmission (E_glu_, +8.25 ± 1.5 mV; *n* = 4 cells) and we recorded IPSCs triggered by auditory nerve stimulation. Then, the membrane potential was hyperpolarized to the reversal potential for chloride ions (E_Cl_, −60 ± 0.7 mV; *n* = 4 cells) to isolate EPSCs evoked by auditory nerve stimulation. We analyzed latencies of EPSCs and IPSCs after the stimulus, as well as the interval between the EPSC and IPSC in the sequence. We refer to the beginning of the stimulation artifact as t0, and to the onsets of EPSC and IPSC as t1 and t2, respectively (Figure 2A, bottom). We found that the timing for activation of the feed-forward inhibitory circuit was very precise with latencies of EPSCs, IPCSs and relative EPSC-IPSC sequences of 1.50 ± 0.16 ms, 2.68 ± 0.13 ms and 1.18 ± 0.09 ms, respectively (*n* = 4 cells, Figure 2B).

### Innervation patterns of fusiform cells and interneurons by auditory nerve fibers

Auditory nerve fibers innervate both fusiform and TVCs (Wickesberg and Oertel, 1988; Zhang and Oertel, 1994; Fujino and Oertel, 2003). However, the pattern of innervation is not known: disynaptic inhibition in FCs may arise from the activation of the same auditory nerve fibers that form synapses onto both the fusiform and the tuberculoventral cell, or fibers innervating FCs may be different from those innervating tuberculoventral cells. Even though evidence from previous anatomical studies exists that some of the fusiform cell targets of TVCs are innervated by the same auditory nerve fibers as the TVCs themselves (Wickesberg and Oertel, 1988), no physiological evidence exists to support this innervation pattern.

**Figure 3:**
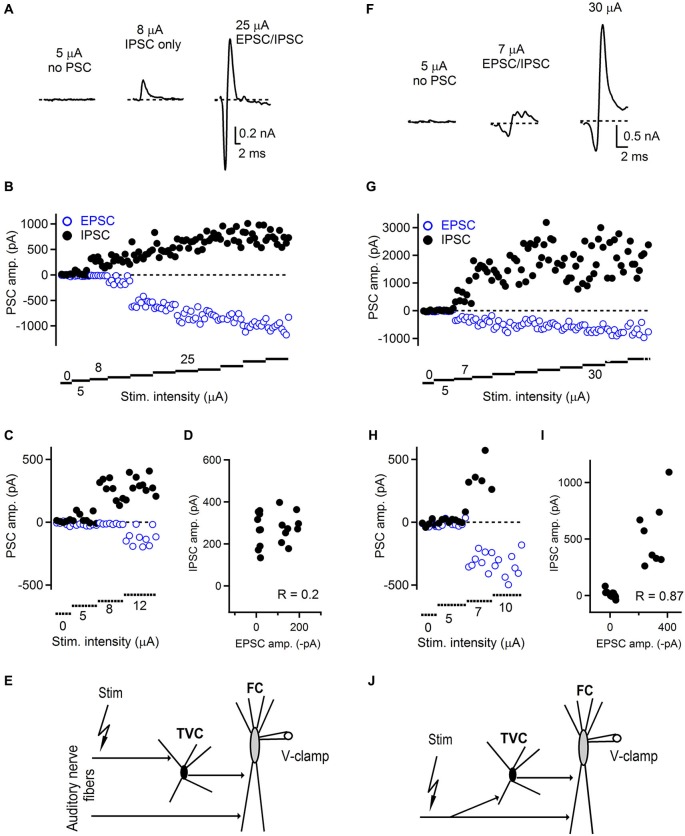
**Innervation pattern of the auditory nerve fibers in the dorsal cochlear nucleus. (A)** Representative examples of feed-forward inhibition evoked by auditory nerve stimulation and recorded from FCs. At low stimulation intensity, no postsynaptic currents were recorded (left trace). When the stimulation was increased, only an IPSC appeared with a latency that was characteristic for a disynaptic connection (middle trace). With further increase of the stimulation intensity, EPSCs appeared while the amplitude of IPSCs increased (right trace) and amplitudes of both components later saturated. Trials that were evoked with the lowest stimulation intensity evoked both successes and failures of synaptic events that can be seen in the plot shown in **(B)**. **(C)** Plot of low stimulation evoked EPSC/IPSC amplitudes shown at expanded scale. **(D)** Plot of IPSC vs. EPSC amplitudes showing low correlation (*R* = 0.2) between IPSCs and EPSCs. **(E)** Schematic drawing of the innervation pattern described in **A–D**. Separate auditory nerve fibers innervate the fusiform (FC) and tuberculoventral (TVC) cells. ~54% (7/13) of FCs tested exhibited this type of innervation. **(F)** Representative examples of FFI and innervation pattern in which the same set of auditory nerve fibers innervates both tuberculoventral and FCs. Following failures in both EPSCs and IPSCs, both components appeared with the same threshold. Stimulation artifacts in **A** and **F** were removed for clarity and all traces represent averages of 5–10 trials. **(G)** With increasing stimulation intensity both components increased in their amplitude until both of them reached saturation. **(H)** Plot of low stimulation evoked EPSC/IPSC amplitudes shown at expanded scale. **(I)** Plot of IPSC vs. EPSC amplitudes showing high correlation (*R* = 0.87) between IPSCs and EPSCs.** (J)** A schematic drawing of the innervation pattern shown in **F–I**. ~46% (6/13) of FCs tested exhibited this type of innervation.

To address this, we recorded FFI in FCs using increasing stimulation intensity to evoke auditory nerve-mediated synaptic responses (Chen and Regehr, 2000; Blitz and Regehr, 2005; Cao and Oertel, 2010; Figure 3). We predicted that if the same set of auditory nerve fibers innervates both the fusiform and the TVCs in the disynaptic circuit, then both the excitatory and inhibitory components would have the same activation threshold. Alternatively, EPSCs and IPSCs could have distinct thresholds, indicative of innervation by different sets of auditory nerve fibers. To distinguish between these possibilities, we used low stimulation intensities (0–25 μA) incremented in 1–5 μA steps to record both EPSC/IPSC failures and successes. As the stimulation intensity increased in subsequent trials, successful synaptic events started to appear (Figure 3). We consider the constant stimulation intensity that gave rise to failures and successes to be the minimal stimulation in these experiments.

We found that in a subset of cells, the threshold for evoking both EPSC and IPSC in the disynaptic sequence was different (Figures 3A–E). In this example, the first successful synaptic events recorded were IPSCs without any EPSCs. The latency of these inhibitory responses corresponded to the disynaptic latencies shown in Figure 2, confirming they were not evoked by direct stimulation. As the stimulation intensity increased, the excitatory component appeared as well. This innervation pattern represented ~54% (7/13 FCs). The remaining ~46% of FCs exhibited an innervation pattern in which thresholds for evoking excitatory and inhibitory components in disynaptic circuit were the same (Figures 3F–J). In this example, both components were recruited at the same level of stimulation intensity. With further increase in the stimulation intensity, the amplitudes of EPSCs remained unchanged, or slightly increased with further stimulation, and the IPSC amplitudes further increased until they reached their maximum value (Figure 3G). We further analyzed the correlation between IPSC/EPSC amplitudes in individual trials to examine whether these are interdependent and possibly activated by the same or different sets of fibers. Figure 3D shows a plot where there is a weak correlation between the IPSC and EPSC amplitudes (*R* = 0.20), whereas in the case of recordings where both EPSCs and IPSCs have the same activation thresholds, strong correlation (*R* = 0.87) between IPSC and EPSC amplitudes was observed (Figure 3I). Several possible explanations exist for the observed results, although clear and precise conclusions about the innervating pattern by the auditory nerve fibers are difficult to draw. Our results show that distinct auditory nerve fibers may innervate fusiform and TVCs separately, or the same fiber can innervate both cell types. However, since there is no direct evidence for innervation by single auditory nerve fibers, due to insufficient resolution of the stimulation technique, our minimal stimulation trials can also represent activation of multiple weak auditory nerve fibers as with the same activation threshold. Challenging experiments such as simultaneous recordings from fusiform and tuberculoventral cells, while stimulating auditory nerve could eventually provide more insight into the issue.

An interesting and important finding of the presented study is the TVC-mediated amount of inhibition onto FCs. In contrast to previously published results (Kuo et al., 2012) describing unitary TVC to FC connections as weak, we show that increasing intensity of stimulation results in large amount of auditory nerve driven inhibition that is received by FCs. In our FFI experiments, we also analyzed the IPSC amplitudes in order to estimate the minimal and maximal number of TVCs innervating a single FC. Our results show that the minimal amplitude of IPSCs when we recorded failures and successes during FFI trials was 163 ± 25 pA (*n* = 10 cells), which corresponds to average conductance 4 nS (range 2–7 nS) at −40 mV. The mean saturating amplitude of IPSCs during same FFI trials was 1594 ± 365 pA (*n* = 10 cells), corresponding to an average conductance of 40 nS (range 9–105 nS) when recorded at −40 mV. Based on the previously reported data on unitary TVC-FC conductance (approximately 2.1 nS at −60 mV) (Kuo et al., 2012), our results suggest that the estimated average number of TVCs innervating a single FC is between 2 (activated by a single auditory nerve fiber) and 20 with saturating stimulation intensity. However, the range of unitary conductances that Kuo et al. (2012) report is 0.7–10.3 nS, as well as the range of conductances reported in the present study mean that the exact and accurate number of TVCs may vary by several fold. One of the explanations of the discrepancy between our study and Kuo et al. (2012) can be divergence of auditory nerve fibers onto TVCs combined with convergence of TVC inputs to FCs, because auditory nerve fibers can activate multiple TVC inputs to FCs and, moreover, multiple TVCs could innervate a single FC. Also, recording and stimulation conditions in the two studies are markedly different which contributes to the differences in the amount of inhibition observed.

### Dynamics of feed-forward inhibitory circuit during repetitive auditory nerve activity

Excitatory synapses formed by the auditory nerve onto various postsynaptic targets in all three subdivisions of the cochlear nucleus exhibit short-term synaptic plasticity with varying amounts of synaptic depression related to their specific postsynaptic target, including FCs in the DCN (Wu and Oertel, 1987; Cao et al., 2008; Yang and Xu-Friedman, 2008; Cao and Oertel, 2010; Chanda and Xu-Friedman, 2010; Kuo et al., 2012). Relatively less is known about the short-term synaptic plasticity of inhibitory inputs that contact basal dendrites of FCs (Kuo et al., 2012). Also, little is known about the temporal dynamics of FFI with both excitation and inhibition intact.

**Figure 4:**
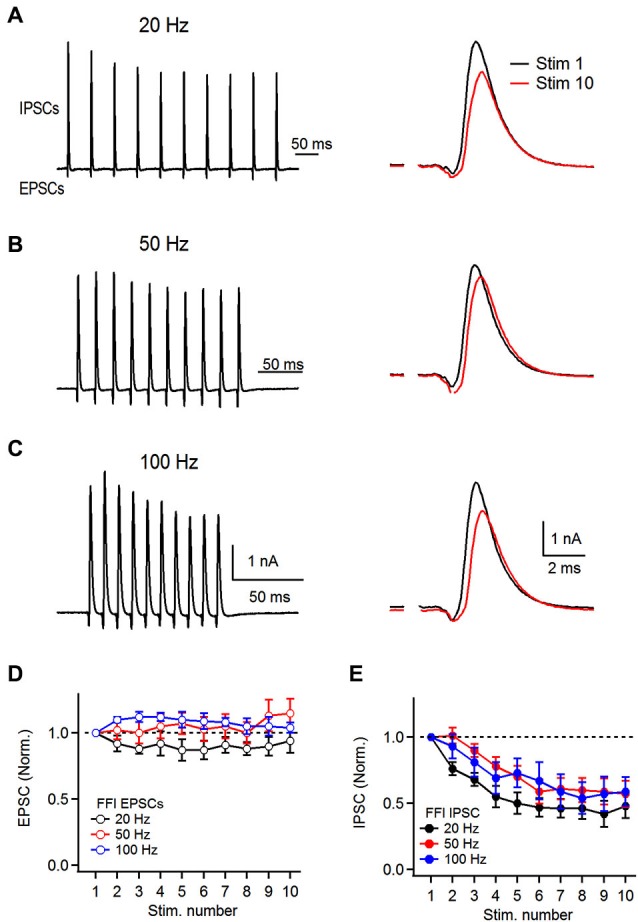
**Dynamics of feed-forward inhibitory circuit during repetitive auditory nerve activity. (A)** Representative recordings of feed-forward inhibition. 10 stimuli were delivered to the auditory nerve at 20 Hz and short-term plasticity of both the excitatory and the inhibitory components was analyzed. Stimulation artifacts were removed for clarity from all traces shown. Response to the first (Stim 1) and 10th (Stim 10) are superimposed on the right. **(B,C)** Recordings of feed-forward inhibition in FCs at 50 and 100 Hz. **(D)** Plot of EPSC short-term plasticity in the EPSC-IPSC sequence at 20, 50 and 100 Hz. Amplitudes were normalized to the first EPSC in the train. **(E)** Plot of IPSC short-term plasticity during FFI trials at 20, 50 and 100 Hz showing pronounced depression. IPSCs were normalized to the first PSC in the train **(B and C**, *n* = 6 cells).

Therefore, to ask how repetitive activity of the auditory nerve affects the FFI onto the FCs, we first recorded mixed excitatory and inhibitory responses in response to stimulus trains delivered to the auditory nerve. FCs were voltage clamped at −40 mV and FFI was evoked by repetitive stimulation of the auditory nerve (10 stimuli) at 20, 50 and 100 Hz. We found that the feed-forward inhibitory circuit associated with basal dendrites of FCs undergoes short-term synaptic plasticity at all frequencies tested (Figure 4). However, plasticity of excitation and inhibition differed. After 10 stimuli delivered to the auditory nerve, the amplitude of the last EPSC in the train was not significantly different from the first EPSC at the frequencies tested (94 ± 9%, 115 ± 11% and 104 ± 4%, at 20, 50, and 100 Hz respectively, Figures 4A–D, *n* = 6 cells). In contrast to the excitation, the inhibitory component of FFI exhibited significant depression at all frequencies tested (S10/S1 of 48 ± 9%, 57 ± 10%, and 59 ± 11% at 20, 50 and 100 Hz stimulation, respectively, Figure 4E). In sum, these results show that repetitive auditory nerve stimulation generates mixed excitatory-inhibitory responses. The excitatory component exhibits little short-term plasticity when activated at 20–100 Hz. In contrast the inhibitory component exhibits moderate depression of 40–50%.

Recently it has been shown that IPSCs evoked in FCs by stimulation of TVCs exhibit mild facilitation when activated at 100 Hz in paired recordings (Kuo et al., 2012), which is in contrast with our observation of IPSC depression. One factor that can influence the amplitudes of EPSCs and IPSCs in FCs during the biphasic response to auditory nerve stimulation is temporal overlap of the synaptic conductances. Although IPSCs are disynaptic and therefore have a longer latency, FCs exhibit relatively slow EPSC kinetics compared to other auditory nerve targets (Gardner et al., 1999) and therefore the inward peak of the biphasic response can be truncated by onset of the fast IPSC. An additional factor that can influence IPSC amplitudes during FFI is plasticity at the auditory nerve to tuberculoventral cell synapse. Either of these factors could explain differences in short-term plasticity of IPSCs when evoked by direct activation of inhibitory axons compared to those evoked by feed-forward activation of interneurons by auditory nerve fibers. We therefore performed experiments to test these possibilities.

**Figure 5:**
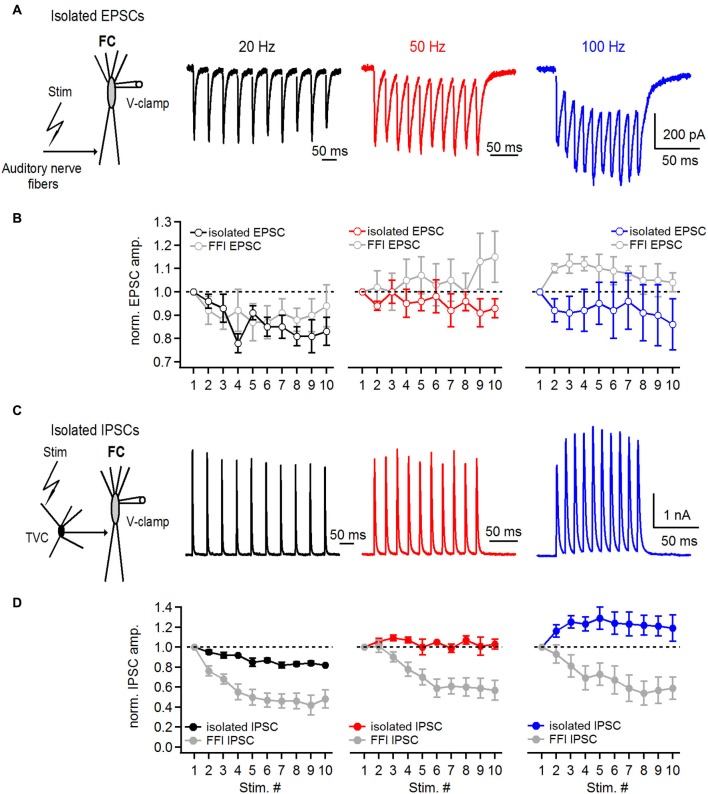
**Short-term plasticity of directly stimulated excitatory and inhibitory inputs onto basal dendrites of fusiform cells. (A)** Representative examples of EPSCs recorded from a fusiform cell evoked by 20, 50 and 100 Hz auditory nerve stimulation. Schematic in the left shows the experimental protocol. Recordings were performed with 2 μM strychnine and 40 μM picrotoxin in perfusion solution. **(B)** Plots of EPSC amplitudes at corresponding stimulation frequencies of the auditory nerve. EPSCs in the train were normalized to the first EPSC in the train. For comparison, gray data points in all three graphs represent data of EPSC short-term plasticity evoked during feed-forward inhibition trials shown in Figure 4 (*n* = 5 cells). **(C)** Representative examples of IPSCs recorded from a fusiform cell evoked by direct stimulation of inhibitory inputs in the deep layer of the DCN at 20, 50 and 100 Hz. Schematic drawing in the left shows the experimental protocol. All recordings were done in 20 μM DNXQ and 5 μM R-CPP to block excitatory transmission. **(D)** Plots of IPSC amplitudes in the train normalized to the first IPSC at corresponding frequencies of stimulation of inhibitory inputs. Gray data of IPSC short-term plasticity recorded during feed-forward inhibition trials and shown in Figure 4 are included for comparison. Differences in short-term plasticity of directly stimulated IPSCs and those evoked during FFI were statistically significant (*P* < 0.001, *n* = 6 cells). All stimulation artifacts in **A and C** were removed for clarity. Traces represent averages of 5–10 trials.

In the first set of experiments we examined short-term plasticity of pharmacologically-isolated excitatory and inhibitory inputs onto FCs. By blocking GABA and glycine receptors (with picrotoxin and strychnine, respectively) we recorded isolated auditory nerve EPSCs evoked at 20–100 Hz. Under conditions where FFI is blocked (Figure 5A), auditory nerve-evoked EPSCs undergo slight depression (S10/S1 of 83 ± 6%, 93 ± 4% and 86 ± 11% at 20, 50 and 100 Hz, respectively, Figures 5A,B, *n* = 5 cells).

Next, we recorded IPSCs from FCs in the presence of DNQX and R-CPP, blockers of excitatory transmission, to investigate whether the amount of IPSC synaptic depression would be the same as during the FFI trials. FCs were voltage clamped at −40 mV and IPSCs were evoked by direct stimulation of inhibitory inputs in the deep layer of the DCN (Figure 5C). Surprisingly, IPSC trains exhibited slight synaptic depression at 20 Hz (S10/S1 was 82 ± 1%), and slight facilitation at 50 and 100 Hz, respectively (S10/S1 at 50 Hz was 103 ± 5%, S10/S1 at 100 Hz was 119 ± 13%; *n* = 6 cells, Figure 5D). These results are consistent with previously reported data from paired recordings between tuberculoventral and FCs (Kuo et al., 2012). However, the comparatively moderate short-term plasticity of directly evoked and pharmacologically isolated IPSCs seems unlikely to explain the pronounced short-term depression of inhibition during FFI when excitation and inhibition are intact.

**Figure 6:**
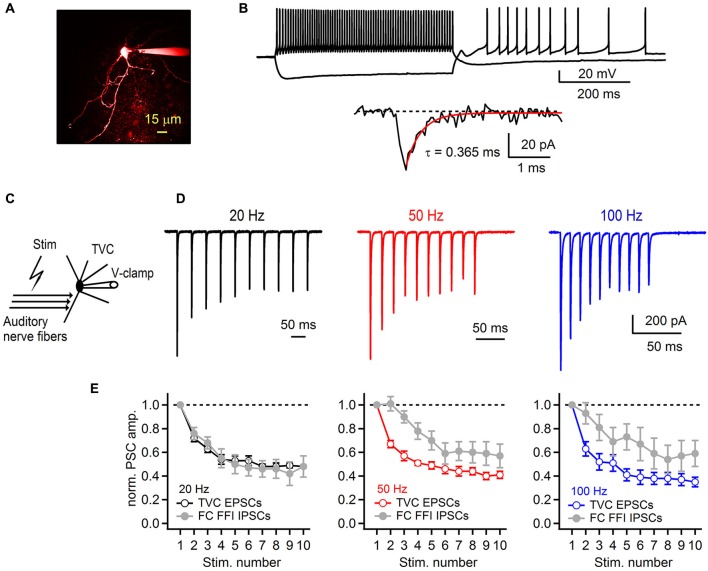
**Synaptic plasticity at the auditory nerve-tuberculoventral cell synapse regulates feed-forward inhibition onto fusiform cells. (A)** A two-photon laser scanning microscopy image of a tuberculoventral cell loaded with Alexa Fluor 594. **(B)** Electrophysiological properties of tuberculoventral cells in DCN slices. Top traces show tonic firing upon prolonged depolarization, high input resistance examined with hyperpolarizing current injection that resulted in rebound firing when returned back to resting membrane potential (−66 mV). Bottom trace shows a representative example of a spontaneous EPSC (sEPSC). Single exponential fit of the rapid decay kinetics is shown in red. **(C)** Experimental protocol for recording auditory nerve evoked EPSCs from tuberculoventral cells. **(D)** Short-term plasticity of EPSCs recorded from a tuberculoventral cell during repetitive auditory nerve stimulation at 20, 50 and 100 Hz. All recordings were performed with 2 μM strychnine and 40 μM picrotoxin in the perfusion solution. Note the pronounced depression that is comparable to the amount of depression of the inhibitory component of FFI sequence recorded from FCs. Stimulation artifacts were removed for clarity. Traces represent averages of 5–10 trials. **(E)** Plots of EPSC amplitudes at corresponding stimulation frequencies of the auditory nerve. Gray data of IPSC short-term plasticity recorded from FCs during feed-forward inhibition trials and shown in Figure 4 are included for comparison. EPSCs in the train were normalized to the first EPSC in the train (*n* = 6 cells).

Therefore, we next investigated whether short-term plasticity at the auditory nerve to tuberculoventral cell synapse accounts for the strong IPSC depression during FFI. For this purpose, we recorded auditory nerve-evoked EPSCs from TVCs visually identified in the slice (Figure 6A). One characteristic of TVCs is that they exhibit fast EPSCs with sub-millisecond decay kinetics (Gardner et al., 1999; Kuo et al., 2012). In our experiments, we confirmed this (Figure 6B) and used the rapidly decaying spontaneous synaptic events as a criteria for distinguishing TVCs from other cell types in the deep layer of the DCN (Gardner et al., 1999). Another distinguishing characteristic is that TVCs are mostly electrically silent (Shofner and Young, 1985; Spirou et al., 1999), and in slices rarely spike spontaneously (Kuo et al., 2012), which we confirmed by recording in cell-attached mode before breaking into the whole-cell configuration (data not shown). Finally, we confirmed the cell identity by inspecting their morphology using fluorescent dye in the recording pipette (Figure 6A). EPSCs recorded from TVCs were evoked by extracellular stimulation of the auditory nerve (Figure 6C), similar to EPSCs recorded from the FCs. Repetitive stimulation of the auditory nerve with 10 stimuli evoked a train of EPSCs that exhibited pronounced short-term synaptic depression (Figure 6D). At all frequencies tested (20, 50 and 100 Hz), EPSCs significantly depressed with S10/S1 of 48 ± 1%, 41 ± 3% and 35 ± 4% at 20, 50 and 100 Hz, respectively (Figure 6E, *n* = 6 cells). These values match very closely with the amount of depression of IPSCs evoked during FFI recorded from the FCs (see Figure 4). Therefore, these results, together with our previous findings strongly indicate that short-term depression at the synapse between the auditory nerve and tuberculoventral cell accounts for the activity dependent change in excitation-inhibition balance in a synapse specific manner. It also accounts for the observed shift in short-term synaptic plasticity of fusiform cell deep-layer inhibition, from facilitation when IPSCs are directly stimulated, to strong depression when FFI is intact.

## Discussion

In the current study, we investigated the properties and mechanisms of FFI in the DCN driven by the auditory nerve. We used patch clamp recordings from fusiform and TVCs to provide evidence that synapse specific and activity dependent synaptic plasticity regulates the balance of excitation and inhibition in a feed-forward inhibitory synaptic circuit associated with basal dendrites of FCs in the DCN.

Our results show that auditory nerve fibers activate both FCs and TVCs that represent the DCN principal neurons and local interneurons, respectively. TVCs further provide strong FFI to FCs. The strong inhibition of FCs that we observe differs from what has recently been shown by Kuo et al. (2012), who demonstrate that connections between TVCs and FCs are rather weak. One explanation that can account for this discrepancy is that the stimulation paradigms between the two studies are different. Paired recordings that Kuo et al. (2012) used in their study represent unitary connections with only a small number of synapses being activated. In case of our experiments, bulk stimulation of the auditory nerve activates multiple auditory nerve fibers that could lead to activation of multiple TVC inputs to FCs. In addition, multiple TVCs could innervate a single FC which would further increase the amount of inhibition recorded from a single FC. A more complete understanding of the circuit mechanisms that control fusiform cell responses to sound will also require understanding of the effects of inhibitory contacts among TVCs themselves (Kuo et al., 2012).

The activation and timing of the disynaptic inhibition that we recorded was fast and very precisely timed, occurring within approximately 1 ms after auditory nerve activation. The narrow time window for summation of inputs has been shown to play a significant role in reaching action potential threshold in postsynaptic neurons (Pouille and Scanziani, 2001), and it is likely to be important in regulating the output of the DCN. Fast and consistently narrow jitter of FFI timing in our experiments provides further evidence for a disynaptic connection. The delay that we describe here is comparable with timing of FFI in the visual system (Blitz and Regehr, 2005), cerebellum (Mittmann et al., 2005) or cortex (Gabernet et al., 2005), but was shorter than in case of FFI described previously in hippocampus (Pouille and Scanziani, 2001; Torborg et al., 2010). Different needs for speed of synaptic transmission and input integration in various brain regions may account for these discrepancies. One of the mechanisms underlying the precision and accurate timing of FFI in the local DCN circuit that we studied may be the kinetics of EPSCs evoked in TVCs by stimulation of auditory nerve fibers. Previous studies (Gardner et al., 1999, 2001), and our data (Figures 6B,D) demonstrate that TVC EPSCs exhibit extremely fast kinetics. The rapid decay kinetics is hypothesized to be predominantly due to expression of fast AMPA receptors containing GluR4 subunits in cells receiving exclusively auditory nerve excitatory inputs (Gardner et al., 1999, 2001).

By using low-intensity stimulation of the auditory nerve we further observe that some auditory nerve fibers evoke a biphasic response in FCs that consists of a monosynaptic EPSC and a disynaptic IPSC. In other cases, EPSCs exhibited a higher threshold for activation and only IPSCs were observed. With increasing stimulation intensity, and thus recruiting more auditory nerve fibers, we observed that excitatory and/or inhibitory components of the FFI began to be recruited gradually and contributed to the overall complex responses recorded from FCs. Similar observations have been described in the visual system (Blitz and Regehr, 2005); however, unlike the mentioned study, we were not always able to distinguish activation of a single auditory nerve fiber from activation of a few weaker fibers in our experiments. Therefore, it is difficult to draw clear conclusions defining the precise innervation pattern of fusiform and TVCs by auditory nerve fibers. Using a challenging approach such as simultaneous recordings from both fusiform and TVCs while stimulating the auditory nerve could help resolve this issue.

Data from *in vivo* experiments show that FCs can be divided into two groups, intensity-selective and intensity-nonselective, based on their responses to sound of various intensities (Zhou et al., 2012). Intensity-selective neurons have a non-monotonic rate-level function and thus respond most strongly to sounds of a specific intensity, whereas intensity non-selective neurons have a monotonic rate-level function. Although the circuit mechanisms for these distinct response types are not known, an intriguing possibility is that differential patterns of FFI by TVCs could play a role. TVCs in DCN are thought to represent type II neurons (Davis and Voigt, 1997; Rhode, 1999) and are important for intensity selectivity of FCs because of their ability to suppress or even eliminate FC firing. Therefore, the innervation patterns of FCs and TVCs can play a crucial role in the output of the DCN since low sound intensities can recruit excitatory inputs onto FCs in the absence of inhibition. Further increases in sound intensity strengthens both excitatory and inhibitory components (Zhou et al., 2012). Our results demonstrate strongly increasing amplitude of inhibition as progressively more auditory nerve fibers are activated, a situation analogous to progressive recruitment of auditory nerve fibers by increasing sound intensity.

An important question related to synaptic input integration and information processing is how repetitive activity regulates the output of a synaptic circuit, especially when both excitatory and inhibitory components are involved. Excitatory synapses between the auditory nerve and its targets have been shown to exhibit various amounts of short-term synaptic depression depending on the type of postsynaptic neuron (Cao and Oertel, 2010; Kuo et al., 2012). Consistent with recent work (Kuo et al., 2012), we observe minimal depression at the auditory nerve-FC synapse and pronounced depression at the synapse between the auditory nerve and TVCs, under conditions where excitation and inhibition, respectively, were pharmacologically isolated. This indicates target-specific specializations of short-term synaptic plasticity, a phenomenon that has also been reported in other brain areas, but is not well understood (Blackman et al., 2013). However, examination of short-term plasticity of EPSCs and IPSCs recorded from FCs during FFI trials provided evidence that short-term plasticity of synaptic currents under conditions when both components are present differs from that recorded when either of the two components is isolated. Such differences may arise from temporal overlap of synaptic conductances during repetitive stimulation. In the case of DCN FCs, the combination of relatively slow EPSC kinetics (Gardner et al., 1999) and fast IPSC kinetics (Xie and Manis, 2013) results in greater summation of excitatory responses during repetitive synaptic activity and IPSC amplitudes are consequently reduced by the tonic inward current. A second factor is synaptic depression at the auditory nerve-tuberculoventral cell synapse (Kuo et al., 2012, our Figure 6). In DCN FCs, synaptic depression at the feed-forward synapse onto the inhibitory interneuron is of greater magnitude than the degree of summation of either excitatory or inhibitory responses during repetitive activation of the feed-forward circuit. In conclusion, our study demonstrates how FFI can regulate the balance of excitation and inhibition in FCs in a dynamic manner during repetitive auditory nerve activity, findings that may be relevant for our understanding of the role of FFI in other brain regions as well.

## Conflict of interest statement

The authors declare that the research was conducted in the absence of any commercial or financial relationships that could be construed as a potential conflict of interest.
